# Ongoing increasing trends in central precocious puberty incidence among Korean boys and girls from 2008 to 2020

**DOI:** 10.1371/journal.pone.0283510

**Published:** 2023-03-22

**Authors:** Sinyoung Kang, Mi Jung Park, Jung Min Kim, Jin-Sung Yuk, Shin-Hye Kim

**Affiliations:** 1 Department of Pediatrics, Samil Hospital, Daegu, Korea; 2 Dr. Park Mijung’s Child Growth Clinic, Seoul, Korea; 3 Department of Internal Medicine, Inje University Sanggye Paik Hospital, Seoul, Korea; 4 Department of Obstetrics and Gynecology, Inje University Sanggye Paik Hospital, Seoul, Korea; 5 Department of Pediatrics, Inje University Sanggye Paik Hospital, Seoul, Korea; Osaka City General Hospital, Children’s Medical Center, JAPAN

## Abstract

**Background:**

Over the last few decades, there has been growing evidence of earlier onset and progression of puberty worldwide. This population-based longitudinal cohort study aimed to analyze the change in the annual incidence rate of central precocious puberty (CPP) among Korean children over the most recent decade, using the national registry data.

**Method:**

The International Statistical Classification of Diseases and Related Health Problems, Tenth Revision (ICD-10) and insurance claims for gonadotropin-releasing hormone agonist (GnRHa) treatment were used to identify CPP patients who were using the Korean Health Insurance Review & Assessment Service (HIRA) database between 2008 and 2020. Patients who began GnRHa therapy before the age of 9 and 10 for girls and boys, respectively, were included in the study.

**Results:**

A total of 6,906 boys and 126,377 girls were diagnosed with CPP between 2008 and 2020. The annual incidence of CPP increased by 83.3 times in boys (from 1.2 to 100 per 100,000 persons) and by 15.9 times in girls (from 88.9 to 1414.7 per 100,000 persons). The age-specific annual incidence of CPP increased remarkably more in older children than in younger ones; the 2020 CPP incidence among 9-year-old boys and 8-year-old girls reached 705.2 and 7,967.3 per 100,000 persons, respectively. The annual prevalence of CPP in boys and girls increased from 2.7 to 206.5 (76.5 times) and from 141.8 to 3439.9 (24.3 times) per 100,000 persons, respectively.

**Conclusion:**

Based on GnRHa treatment insurance claims, our study suggests that the annual incidence of CPP has substantially increased in Korea during the past 13 years. These findings highlight the importance of meticulous judgment by doctors in determining GnRHa treatment.

## Introduction

Precocious puberty is traditionally defined as the appearance of secondary sexual characteristics before the age of 8 in girls and 9 in boys, as well as the onset of menstruation before the age of 9.5 [1]. The onset of puberty can be assessed by breast enlargement in girls and testicular enlargement in boys, as well as the activation of the hypothalamic-pituitary-gonadal (HPG) axis, detected by laboratory tests [2]. If early activation of HPG axis causes precocious puberty, the disorder is GnRH-dependent and referred to as central precocious puberty (CPP) [3].

There has been a global trend in which pubertal development has been accelerated since the mid-1900s, although the pubertal timing and tempo varied by race and ethnicity [4]. However, after the early 2000s, epidemiologic studies suggested that the pubertal timing and specific age at menarche in girls stabilized in several European nations [5–9]. In the US, despite the fact that the age of breast development among girls had decreased significantly over the past half-century, the age at menarche had decreased by only 2.5 to 4 months over a similar period, indicating that the advancement of pubertal onset in the US population is not accompanied by HPG axis activation [6]. On the other hand, a recent large population-based study indicated that the age of menarche has declined by approximately five months over the last 15 years in Korean girls, which is a much more significant change as compared to other Western countries [10]. This trend of accelerated pubertal development in adolescents was also observed in several countries, including Korea, Sweden, and Denmark, not just in girls but also in boys [11–14].

Few studies have been conducted to examine the prevalence of precocious puberty worldwide, as compared to several studies on normal pubertal timing and its secular changes among adolescents. Studies in Spain [15], Denmark [16], and Korea have reported an increase in CPP incidence. In prior research, we demonstrated that the incidence of CPP among Korean children aged less than 8 (in girls) or 9 (in boys) rose almost 15-fold between 2004 and 2010 [11]. A follow-up study from another group indicated that the increase in Korean CPP incidence was approximately 5-fold in girls and 9-fold in boys for the pediatric population aged less than 9 (in girls) or 10 years (in boys) between 2008 and 2014 [17]. Identifying the long-term trends in overall CPP cases under GnRHa treatment in the real world is essential to estimate the medical and societal burden of CPP. However, given the accelerating pubertal tempo worldwide, it is particularly crucial to evaluate CPP trends by age group in order to predict future implications on public health and to make medical decisions based on treatment targets.

In this study, we aimed to examine the most up-to-date incidences of CPP in Korea and to determine if there was a difference in CPP trends based on sex and age group.

## Materials and methods

### Subjects

#### Study samples

The vast majority of South Korean citizens (97%) are registered with a National Health Insurance Service (NHIS) and are offered insurance coverage for their medical expenses. Korean patients’ diagnoses from hospitals are recorded using the International Classification of Diseases, Tenth Revision (ICD-10) coding system for health insurance claims. The Korea Health Insurance Review and Assessment Service (HIRA) performs reviews of medical documentation for all NHIS-sponsored medical expenses to monitor the adequacy of diagnosis and treatment. Therefore, HIRA data provides a credible source of information for monitoring the incidence and prevalence of uncommon illnesses in the Korean population [18].

The HIRA allows insurance claims for GnRHa treatment only if a diagnosis of CPP is confirmed in accordance with the Korean clinical guidelines for CPP [19]: 1) an emergence of secondary sexual characteristics before age 8 in girls (Breast Tanner stage 2) and before age 9 in boys (Genitalia Tanner 2 stage), 2) advancement of bone age and acceleration of growth, and 3) a pubertal response (a peak luteinizing hormone level ≥ 5 IU/L) after a GnRH stimulation test. Of note, the HIRA sets an upper age limit at which it allows insurance claims for GnRHa therapy, which is 9 years of age in girls and 10 years in boys, to consider a lag period between the first detection of pubertal signs and diagnosis by a physician. To estimate the incidence of CPP in this study, we included CPP children (girls aged <9, boys aged <10) who registered with an ICD-10 diagnostic code for precocious puberty (E22.9 or E30.1) at the HIRA database as starting GnRHa treatment for the first time between January 1, 2008, and November 30, 2020. Due to the method used in this study to define CPP cases, it is important to note that the number of CPP cases in this study is not the direct number of cases determined from medical records, including medical histories and laboratory data, but a proxy number of CPP cases. Since the NHIS covers GnRHa treatment claims in CPP girls under the age of 12 and in CPP boys under the age of 13, we calculated prevalence by including CPP patients who had received GnRHa therapy within these age limits. Both the incidence and prevalence were determined in terms of units per 100,000 people. Throughout the study period, neither the Korean guidelines for CPP diagnosis nor the HIRA practice for GnRHa insurance coverage changed [19].

The Institutional Review Board at Inje University Sanggye Paik Hospital waived their review board’s approval requirement for this study (approval number: SGPAIK2021-07-001), because the HIRA dataset complies with South Korea’s Bioethics and Safety Act by using anonymous identifying codes to protect personal information. No informed consent was necessary.

#### Incidence and prevalence calculations

The incidence rate was estimated by taking the number of population at risk in accordance with the census in each calendar year, sex, and age as denominators and the number of CPP patients who first started GnRHa treatment in each calendar year, sex, and age as numerators. The number of at-risk population was collected from the National Institute of Statistics of Korea [20]. The prevalence was also calculated using the number of populations at risk in each calendar year, sex, and age as denominators and the number of CPP patients receiving GnRHa treatment in each calendar year, sex, and age as the numerator. 95% confidence intervals of incidence and prevalence estimates were generated from the asymptotic convergence of incidence estimates to the normal distribution under the assumption that the number of CPP follows the Poisson distribution. A generalized linear model (GLM) with Poisson distribution was used to evaluate the relationship of year, sex, and age with the incidence rate of CPP. All statistical data were analyzed using R 4.0.2 (The R Foundation for Statistical Computing, Vienna, Austria) and a two-tailed test. A p-value of less than 0.05 was considered statistically significant.

## Results

### The incidence of central precocious puberty

**Fig 1:**
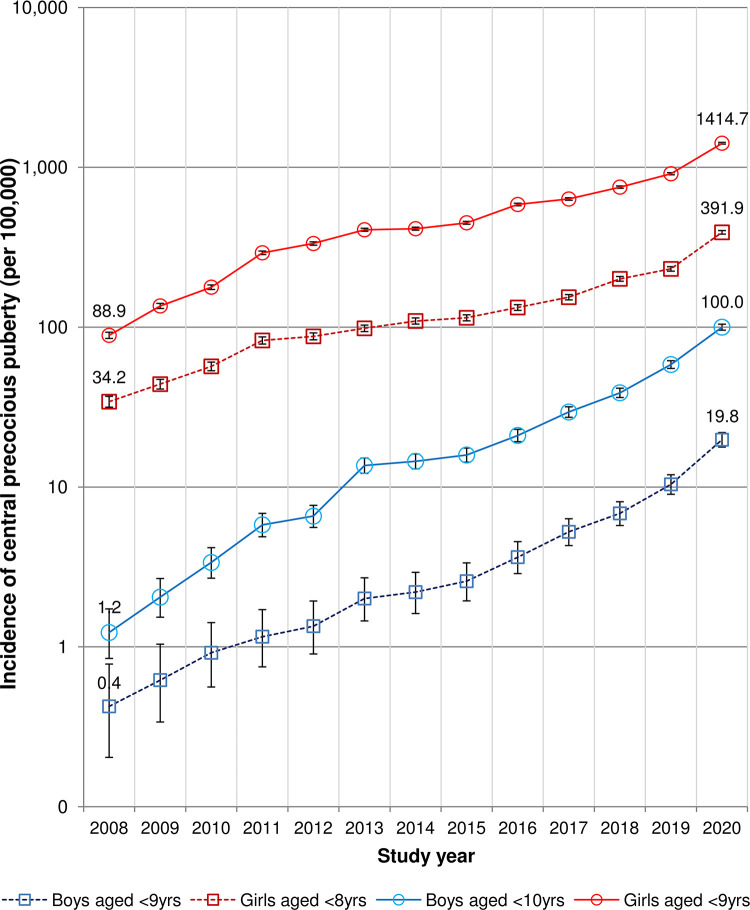
The annual incidence of central precocious puberty. The error bars show the 95 percent confidence intervals for the incidence estimates.

A total of 6,906 boys aged 0–9 years and 126,377 girls aged 0–8 years were diagnosed with CPP between 2008 and 2020, and the incidence of CPP was 489.3 per 100,000 girls and 22.4 per 100,000 boys (Table 1). The annual incidence of CPP among Korean children by sex and diagnostic age cutoffs is shown in Fig 1. The annual incidence of CPP among boys grew by 83.3 times (from 1.2 to 100 per 100,000 persons), while the incidence among girls increased by 15.9 times (from 88.9 to 1414.7 per 100,000 persons). When a stricter diagnostic age cutoff (under 8 and 9 years of age for girls and boys, respectively) was implemented, similar but rather gradual trends were observed, with a 49.5-fold rise in the annual incidence of CPP in boys from 0.4 to 19.8 per 100,000 and a 12-fold increase in girls from 15.4 to 187.7 per 100,000.

**Table 1:** The annual incidence (95% confidence interval) of central precocious puberty per calendar year.

	Total			Boys			Girls		
Year	Cases (n)	Population at risk	Incidence (95% CI)	Cases (n)	Population at risk	Incidence (95% CI)	Cases (n)	Population at risk	Incidence (95% CI)
Diagnostic age limit: under 8 years for girls, under 9 years for boys^(a^^)^									
2008	652	4,234,816	15.4 (14.2–16.6)	10	2,356,475	0.4 (0.2–0.8)	642	1,878,341	34.2 (31.6–36.9)
2009	811	4,071,899	19.9 (18.6–21.3)	14	2,260,326	0.6 (0.3–1.0)	797	1,811,573	44.0 (41.0–47.2)
2010	1,032	3,959,059	26.1 (24.5–27.7)	20	2,178,544	0.9 (0.6–1.4)	1,012	1,780,515	56.8 (53.4–60.5)
2011	1,499	3,943,669	38.0 (36.1–40.0)	25	2,158,407	1.2 (0.7–1.7)	1,474	1,785,262	82.6 (78.4–86.9)
2012	1,593	3,937,511	40.5 (38.5–42.5)	29	2,152,164	1.3 (0.9–1.9)	1,564	1,785,347	87.6 (83.3–92.1)
2013	1,813	3,942,568	46.0 (43.9–48.2)	43	2,142,599	2.0 (1.5–2.7)	1,770	1,799,969	98.3 (93.8–103.0)
2014	2,016	3,938,138	51.2 (49.0–53.5)	47	2,136,605	2.2 (1.6–2.9)	1,969	1,801,533	109.3 (104.5–114.2)
2015	2,106	3,929,148	53.6 (51.3–55.9)	55	2,136,499	2.6 (1.9–3.4)	2,051	1,792,649	114.4 (109.5–119.5)
2016	2,418	3,878,314	62.3 (59.9–64.9)	77	2,114,458	3.6 (2.9–4.6)	2,341	1,763,856	132.7 (127.4–138.2)
2017	2,772	3,790,186	73.1 (70.4–75.9)	108	2,059,586	5.2 (4.3–6.3)	2,664	1,730,600	153.9 (148.1–159.9)
2018	3,507	3,683,375	95.2 (92.1–98.4)	137	2,002,878	6.8 (5.7–8.1)	3,370	1,680,497	200.5 (193.8–207.4)
2019	3,901	3,533,520	110.4 (107–113.9)	201	1,934,485	10.4 (9.0–11.9)	3,700	1,599,035	231.4 (224.0–239.0)
2020	6,270	3,339,896	187.7 (183.1–192.4)	362	1,832,181	19.8 (17.8–21.9)	5,908	1,507,715	391.9 (381.9–402.0)
Overall	30,390	50,182,099	60.6 (59.9–61.2)	1,128	27,465,207	4.1 (3.9–4.4)	29,262	22,716,892	128.8 (127.3–130.3)
Diagnostic age limit: under 9 years for girls, under 10 years for boys^(b)^									
2008	1,971	4,863,302	40.5 (38.8–42.4)	33	2,683,310	1.2 (0.8–1.7)	1,938	2,179,992	88.9 (85.0–92.9)
2009	2,903	4,689,451	61.9 (59.7–64.2)	53	2,590,445	2.0 (1.5–2.7)	2,850	2,099,006	135.8 (130.8–140.9)
2010	3,693	4,521,517	81.7 (79.1–84.4)	84	2,492,030	3.4 (2.7–4.2)	3,609	2,029,487	177.8 (172.1–183.7)
2011	6,032	4,448,927	135.6 (132.2–139)	141	2,430,629	5.8 (4.9–6.8)	5,891	2,018,298	291.9 (284.5–299.4)
2012	6,891	4,423,286	155.8 (152.1–159.5)	158	2,404,884	6.6 (5.6–7.7)	6,733	2,018,402	333.6 (325.7–341.6)
2013	8,526	4,410,300	193.3 (189.2–197.5)	326	2,394,565	13.6 (12.2–15.2)	8,200	2,015,735	406.8 (398–415.7)
2014	8,667	4,383,506	197.7 (193.6–201.9)	343	2,368,402	14.5 (13–16.1)	8,324	2,015,104	413.1 (404.3–422.1)
2015	9,457	4,384,926	215.7 (211.3–220.1)	375	2,365,501	15.9 (14.3–17.5)	9,082	2,019,425	449.7 (440.5–459.1)
2016	12,216	4,358,367	280.3 (275.3–285.3)	495	2,355,998	21.0 (19.2–22.9)	11,721	2,002,369	585.4 (574.8–596.1)
2017	13,045	4,264,138	305.9 (300.7–311.2)	681	2,312,436	29.4 (27.3–31.7)	12,364	1,951,702	633.5 (622.4–644.8)
2018	15,159	4,138,035	366.3 (360.5–372.2)	869	2,237,720	38.8 (36.3–41.5)	14,290	1,900,315	752.0 (739.7–764.4)
2019	18,009	4,004,644	449.7 (443.2–456.3)	1,265	2,167,980	58.3 (55.2–61.7)	16,744	1,836,664	911.7 (897.9–925.6)
2020	26,714	3,824,600	698.5 (690.1–706.9)	2,083	2,083,542	100.0 (95.7–104.4)	24,631	1,741,058	1414.7 (1397.1–1432.5)
Overall	133,283	56,714,999	235.0 (233.7–236.3)	6,906	30,887,442	22.4 (21.8–22.9)	126,377	25,827,557	489.3 (486.6–492.0)

CI, confidence interval.

^(a)^ Population at risk: Girls aged from 0 to 7 years and 364 days, boys aged from 0 to 8 years and 364 days.

^(b)^ Population at risk: Girls aged from 0 to 8 years and 364 days, boys aged from 0 to 9 years and 364 days.

### Age-specific incidence of central precocious puberty

**Fig 2:**
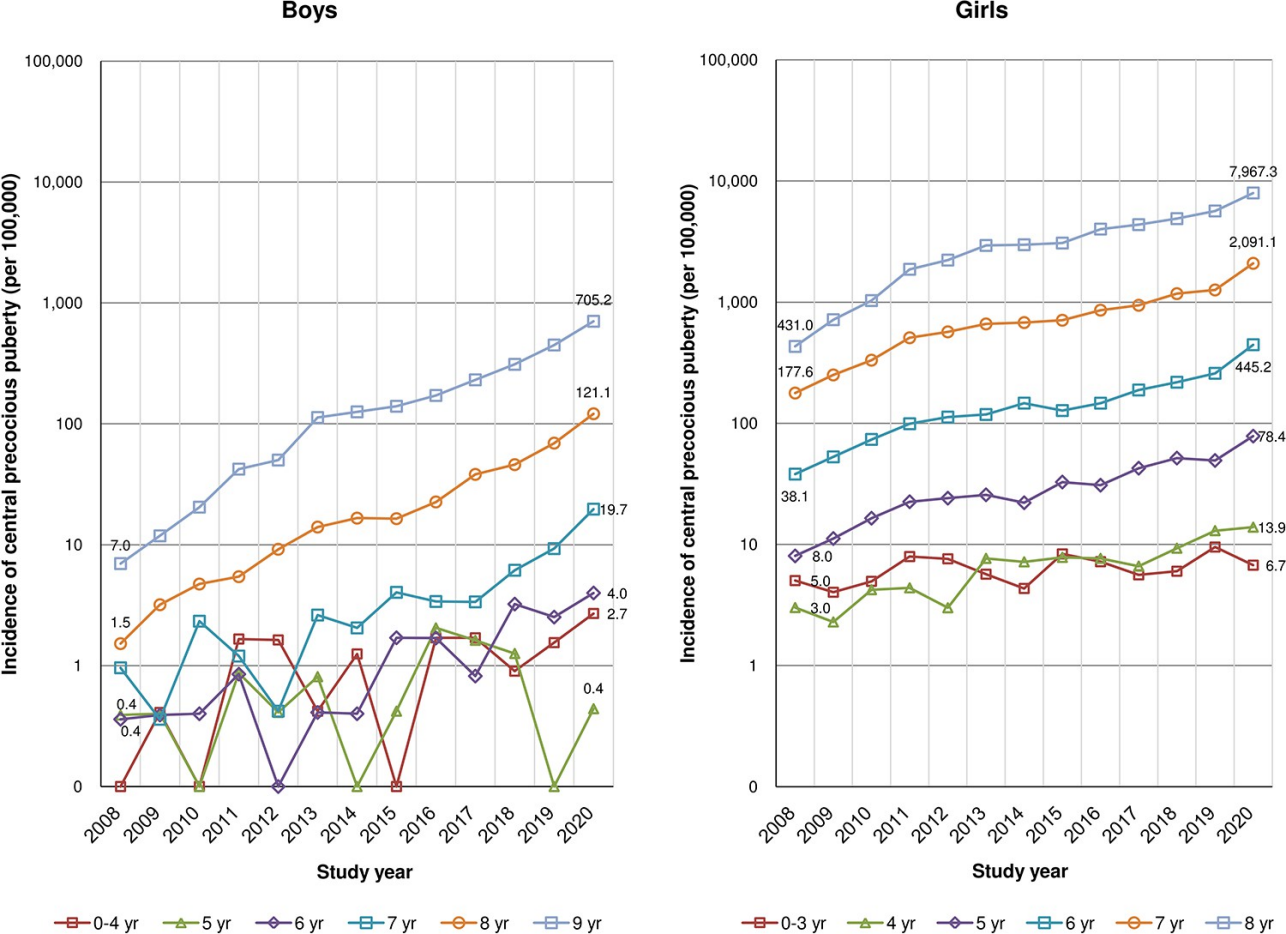
The annual incidence of central precocious puberty according to sex and age at diagnosis.

Fig 2 and S1 Table show the annual incidence of CPP based on sex and age at diagnosis. The age-specific annual incidence of CPP increased significantly in all age groups, with the exception of boys aged 5 years and girls aged 0–3 years. In 2020, the incidence of CPP was greatest among 9-year-old boys and 8-year-old girls at 705.2 and 7,967.3 per 100,000 individuals, respectively (Fig 2, S1 Table). Table 2 illustrates the estimated annual change in central precocious puberty incidence rates by sex and age group. CPP incidence increased by 135.8% per year in boys and 118.5% per year in girls between 2008 and 2020. The age-specific annual incidence of CPP increased considerably with older age groups, with the highest rise occurring in 8-year-old boys (138.9%) and in 8-year-old girls (118.9%).

**Table 2:** The estimated change of central precocious puberty incidence rates per year (IRR and 95% CI) between 2008 and 2020 based on sex and age group.

Boys				Girls			
Age group	IRR	95% CI	P-value	Age group	IRR	95% CI	P-value
0-4yr	1.119	(1.013–1.235)	0.027	0-3yr	1.018	(0.979–1.059)	0.375
5y	1.071	(0.953–1.204)	0.247	4y	1.131	(1.087–1.177)	<0.001
6y	1.256	(1.135–1.389)	<0.001	5y	1.161	(1.139–1.183)	<0.002
7y	1.323	(1.247–1.405)	<0.001	6y	1.176	(1.166–1.187)	<0.003
8y	1.389	(1.354–1.424)	<0.001	7y	1.174	(1.170–1.178)	<0.004
9y	1.358	(1.345–1.371)	<0.001	8y	1.189	(1.187–1.191)	<0.005
0–9 yr	1.358	(1.346–1.369)	<0.001	0–8 yr	1.185	(1.183–1.187)	<0.001

IRR, incidence rate ratio; CI, confidence interval.

**Fig 3:**
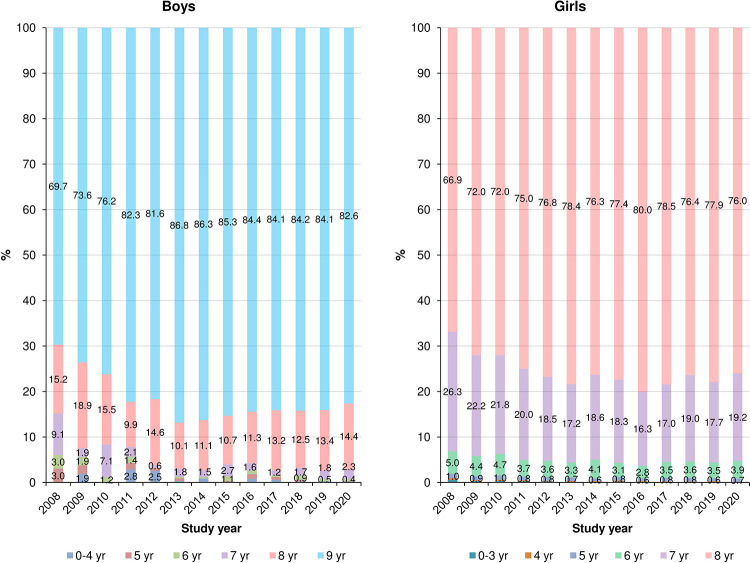
Age distribution of central precocious puberty patients according to sex and calendar year.

Fig 3 depicts the age distribution of CPP cases according to sex and calendar year. Between 2008 and 2013, the proportion of 8-year-old CPP girls increased from 66.9% to 78.4%; however, this proportion remained consistent after 2013. Similarly, the proportion of CPP boys aged 9 years increased from 69.7% to 86.8% between 2008 and 2013, but the proportion of CPP cases based on age remained stable after 2013.

### The prevalence of CPP

**Fig 4:**
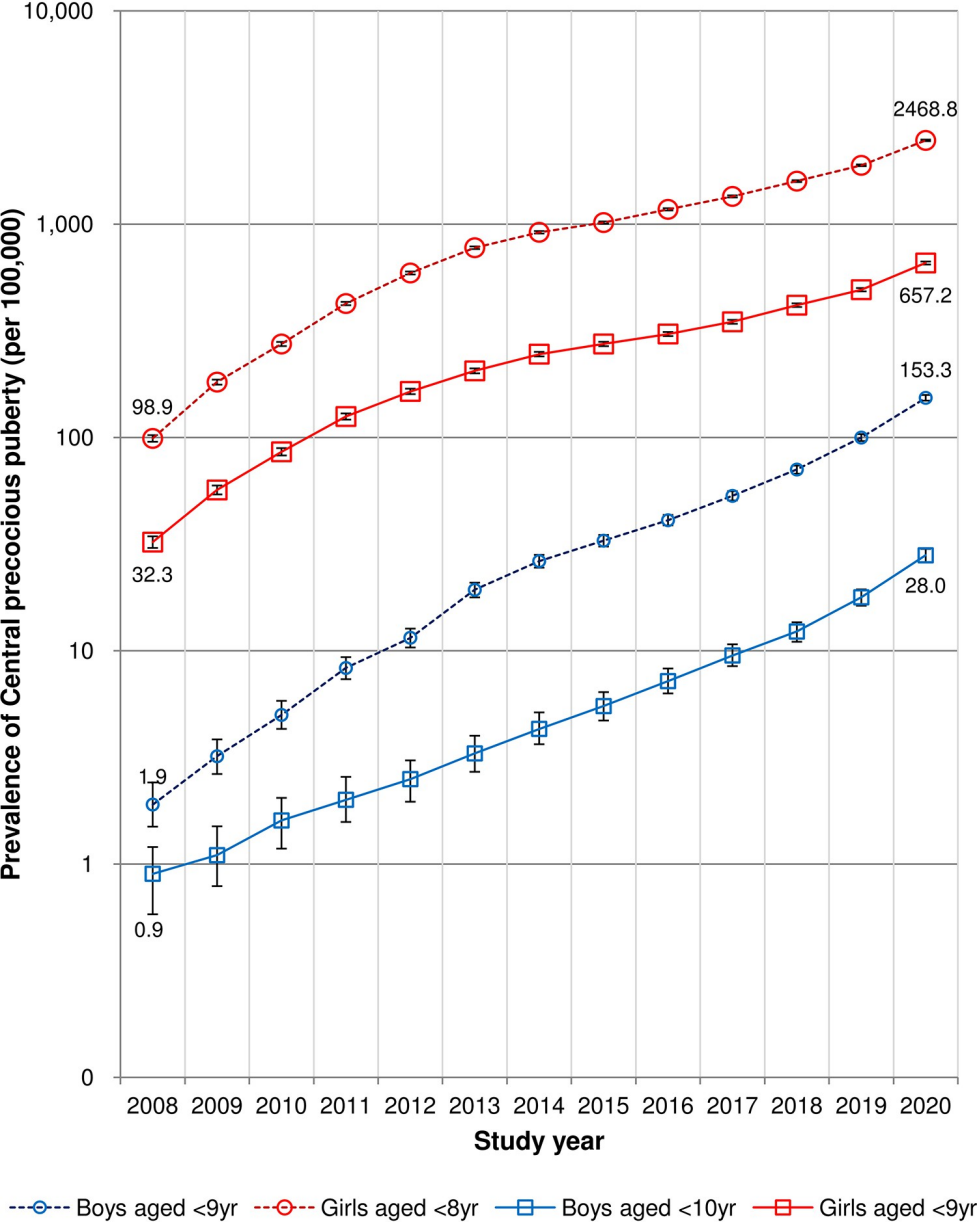
The annual prevalence of central precocious puberty according to sex, calendar year, and two diagnostic age limits. The error bars show the 95 percent confidence intervals for the prevalence estimates.

The annual prevalence of CPP based on sex, calendar year, and two diagnostic age limits is shown in Table 3 and Fig 4. Between 2008 and 2020, the overall prevalence of CPP was 6.8 per 100,000 boys and 250.6 per 100,000 girls. Similar to the trend in incidence, the prevalence of CPP had increased annually and reached 28.0 per 100,000 boys and 657.2 per 100,000 girls by 2020. Between 2008 and 2013, the CPP prevalence rose more sharply than between 2013 and 2020. CPP prevalence increased 31.1 times in boys over 13 years, while it grew 20.3 times in girls. It increases more among boys than girls, similar to the incidence rate (Fig 4).

**Table 3:** The prevalence (95% confidence interval) of central precocious puberty according to the sex, calendar year, and two diagnostic age limits.

	Total			Boys			Girls		
Year	Cases (n)	Population at risk	Prevalence (95% CI)	Cases (n)	Population at risk	Prevalence (95% CI)	Cases (n)	Population at risk	Prevalence (95% CI)
Diagnostic age limit: under 8 years for girls, under 9 years for boys^(a)^									
2008	1,042	6,888,669	15.1 (14.2–16.1)	32	3,761,971	0.9 (0.6–1.2)	1,010	3,126,699	32.3 (30.3–34.4)
2009	1,755	6,651,527	26.4 (25.1–27.7)	40	3,627,505	1.1 (0.8–1.5)	1,715	3,024,022	56.7 (54.0–59.5)
2010	2,566	6,439,739	39.8 (38.3–41.4)	55	3,507,966	1.6 (1.2–2.0)	2,511	2,931,773	85.6 (82.3–89.1)
2011	3,655	6,271,255	58.3 (56.4–60.2)	69	3,409,857	2 (1.6–2.6.0)	3,586	2,861,399	125.3 (121.2–129.6)
2012	4,676	6,124,265	76.4 (74.1–78.6)	82	3,328,342	2.5 (2.0–3.1)	4,594	2,795,923	164.3 (159.5–169.2)
2013	5,721	5,977,010	95.7 (93.2–98.3)	107	3,239,479	3.3 (2.7–4.0)	5,614	2,737,532	205.1 (199.7–210.6)
2014	6,777	5,857,297	115.7 (112.9–118.6)	137	3,154,696	4.3 (3.6–5.1)	6,640	2,702,601	245.7 (239.8–251.8)
2015	7,525	5,789,492	130.0 (127.0–133.0)	171	3,107,093	5.5 (4.7–6.4)	7,354	2,682,399	274.2 (267.9–280.7)
2016	8,344	5,729,362	145.6 (142.4–148.8)	222	3,069,737	7.2 (6.3–8.3)	8,122	2,659,625	305.4 (298.7–312.1)
2017	9,450	5,648,457	167.3 (163.8–170.8)	288	3,018,311	9.5 (8.5–10.7)	9,162	2,630,146	348.3 (341.0–355.7)
2018	11,146	5,549,622	200.8 (197.0–204.7)	363	2,962,363	12.3 (11.0–13.6)	10,783	2,587,259	416.8 (408.9–425.1)
2019	12,914	5,415,675	238.5 (234.2–242.7)	515	2,899,219	17.8 (16.3–19.4)	12,399	2,516,456	492.7 (483.8–501.6)
2020	16,729	5,232,371	319.7 (314.6–324.8)	785	2,806,476	28.0 (26.0–30.0)	15,944	2,425,895	657.2 (646.7–667.8)
Overall	92,300	77574737.5	119.0 (118.1–119.8)	2,866	41,893,012	6.8 (6.6–7.1)	89,434	35,681,726	250.6 (248.9–252.4)
Diagnostic age limit: under 9 years for girls, under 10 years for boys^(b)^									
2008	3,164	6,888,669	45.9 (44.3–47.6)	72	3,761,971	1.9 (1.5–2.4)	3,092	3,126,699	98.9 (95.4–102.5)
2009	5,610	6,651,527	84.3 (82.1–86.6)	116	3,627,505	3.2 (2.6–3.8)	5,494	3,024,022	181.7 (176.8–186.6)
2010	8,211	6,439,739	127.5 (124.7–130.3)	176	3,507,966	5.0 (4.3–5.8)	8,035	2,931,773	274.1 (268–280.4)
2011	12,425	6,271,255	198.1 (194.6–201.7)	283	3,409,857	8.3 (7.4–9.3)	12,142	2,861,399	424.3 (416.7–432.0)
2012	16,881	6,124,265	275.6 (271.2–280.1)	382	3,328,342	11.5 (10.4–12.7)	16,499	2,795,923	590.1 (580.7–599.6)
2013	21,839	5,977,010	365.4 (360.3–370.5)	624	3,239,479	19.3 (17.8–20.8)	21,215	2,737,532	775.0 (764.1–785.8)
2014	25,574	5,857,297	436.6 (430.9–442.3)	829	3,154,696	26.3 (24.5–28.1)	24,745	2,702,601	915.6 (903.7–927.5)
2015	28,297	5,789,492	488.8 (482.9–494.6)	1,019	3,107,093	32.8 (30.8–34.9)	27,278	2,682,399	1,016.9 (1,004.7–1,029.1)
2016	32,455	5,729,362	566.5 (560.2–572.7)	1,257	3,069,737	40.9 (38.7–43.3)	31,198	2,659,625	1,173.0 (1,158.9–1,187.1)
2017	37,082	5,648,457	656.5 (649.3–663.7)	1,607	3,018,311	53.2 (50.6–56.0)	35,475	2,630,146	1,348.8 (1,333.9–1,363.6)
2018	43,240	5,549,622	779.2 (771.4–786.9)	2,093	2,962,363	70.7 (67.6–73.8)	41,147	2,587,259	1,590.4 (1,574.5–1,606.3)
2019	50,351	5,415,675	929.7 (921.4–938.1)	2,894	2,899,219	99.8 (96.1–103.6)	47,457	2,516,456	1,885.9 (1,868.9–1,904.7)
2020	64,194	5,232,371	1,226.9 (1,217.0–1,236.7)	4,303	2,806,476	153.3 (148.7–158.1)	59,891	2,425,895	2,468.8 (2,449.1–2,491.0)
Overall	349,323	77,574,738	450.3 (448.5–452.1)	15,655	41,893,012	37.4 (36.8–38.0)	333,668	35,681,726	935.1 (931.4–938.9)

CI, confidence interval.

^(a)^ Age at diagnosis: Girls aged from 0 to 7years and 364days, boys aged from 0 to 8years and 364days.

^(b)^ Age at diagnosis: Girls aged from 0 to 8years and 364days, boys aged from 0 to 9years and 364days.

## Discussion

In this study, we found that Korean CPP incidence has accelerated in the past 13 years. The annual incidence of CPP increased by 83.3 times in boys (from 1.2 to 100 per 100,000 persons) and by 15.9 times in girls (from 88.9 to 1,144.7 per 100,000 persons), substantially more in older children than in younger children; the incidence of CPP increased most remarkably in boys and girls aged 8 years. Since CPP incidences increased across all age groups, the age distribution of CPP incidence remained constant after 2013. The prevalence of CPP followed a similar pattern to the incidence.

Precocious puberty is conventionally defined as the development of secondary sexual characteristics in girls and boys before the age of 8 and 9 years, respectively, in most countries. This criterion was established based on the old articles on the age of pubertal events from the 1940s [21]. In 1997, in response to the concern that this criterion might be outdated, large-scale national surveys on the pubertal age were conducted in the US [22]. These studies indicated that the mean age of pubertal onset in girls had been advanced as compared to prior studies (9.96 ± 1.82 years in white girls, 8.87 ± 1.93 years in African-American girls). Accordingly, the age of 7 years for white girls and 6 years for African-American girls has been proposed to define precocious puberty [23], assuming no symptoms or signs of a central nervous system disorder or other concurrent illnesses that could also contribute to sexual precocity. However, the conventional diagnostic age limit is still used in the majority of studies on precocious puberty, mainly due to the absence of nationally representative survey data on pubertal development. Furthermore, because of the delay between the initial detection of pubertal development and the clinically verified diagnosis of precocious puberty, several studies and clinicians use the age of under 9 years for girls and under 10 years for boys as a diagnostic age limit for precocious puberty [16,17,24–26].

There have been few studies on the national incidence of CPP. In a prior study, we reported a substantial increase in CPP incidence among Korean girls from 3.3 to 50.4 per 100,000 persons between 2004 and 2010 using the HIRA database [11]. A Spanish study conducted between 2000 and 2009 using clinical data from pediatric endocrinology units also showed an increasing trend of CPP among girls, but the overall incidence of Korean CPP between 2004 and 2010 was more than 20 times higher compared with Spanish CPP incidence between 1997 and 2009 (23.3 vs. 1.1 per 100,000 persons) [15]. Among Korean boys, between 2004 and 2010 the increase in CPP incidence was gradual, from 0.3 to 1.2 per 100,000 boys, and the increment was modest among girls aged < 6 years and boys aged < 7 years. In this study, we showed that the increasing secular trend in Korean CPP was accelerated over the last 13 years in both sexes and that the incidence rise was larger in boys than in girls, contrary to our earlier findings. Recently, a Danish study using national patient registry data likewise reported that the CPP incidence increased more rapidly in boys (15-fold) than in girls (6-fold) between 1998 and 2017, although the rates of CPP increase in our study were 5 to 9 times higher than those reported in Denmark in 2017 [16].

No research has previously explored the trend in CPP incidence by age in detail. In this study, we observed that CPP incidence has grown across almost all age groups. This observation and our recent report on a significant decrease in menarche age in Korean girls [10] support the notion that activation of the HPG axis is associated with earlier pubertal onset in Korean girls. The earlier onset of puberty among Korean children differs from that of US children, in whom pubertal onset was accelerated but not accompanied by HPG axis activation [24,27], the age at menarche remained constant, and the tempo of puberty was slowed [28]. We hypothesize that changes in CPP incidences over the last decade could be attributed to a general shift to the left in pubertal onset and tempo rather than an increase in pathological CPP [29], which is supported by the fact that CPP incidences have increased over the majority of age groups, with the incidence among 8-year-old girls reaching 7.96% in 2020.

Another important finding in our study is that GnRH agonist treatment starting at the age of 8 accounts for 75 to 90% of all cases. Despite there being no conclusive evidence that GnRHa treatment beyond the age of 7 years improves females’ adult height [30–32], GnRHa treatment has been initiated in many children ≥ 8 years old with CPP in Korea, likely reflecting severe parental concerns regarding premature menarche and children’s psychosocial health [33]. Other possible explanations might be vigorous marketing by GnRHa pharmaceutical companies, high accessibility to the healthcare system, and relatively affordable medical costs in Korea [33,34]. According to the Korean Pediatric Endocrinology society’s clinical guidelines, clinicians are recommended to regularly follow up on patients suspected to have CPP for at least 3–4 months to confirm if their symptoms and signs are rapidly progressing before they start GnRHa treatment [19]. Through this process, it is possible to prevent non-progressive CPP patients who do not need treatment from starting treatment. Additionally, it is necessary to establish the normative data of pubertal onset and tempo in Korean children, which estimated to have been accelerated in the last 20 years among the entire population, and to educate physicians and parents to improve their understanding of the normative data in Korea.

We found that the proportions of 8-year-old CPP girls and 9-year-old CPP boys increased considerably between 2009 and 2013; however, this trend halted after 2013. We previously discovered that the incidence of CPP in Korea increased significantly between 2004 and 2010, but primarily among children over the age of six [11]. We had hypothesized that a rise in parental knowledge of CPP, which led to an increase in treatment, was one of the plausible causes for this trend. If parental knowledge was the key reason for the trends in rising Korean CPP incidence in this study, the proportion of CPP children aged 8–9 years should have continuously increased, whereas CPP incidence among children aged 6 would have shown just a modest increase. In light of our new results on the age-specific incidence and proportions of CPP, parental awareness and CPP screening practices may have contributed to the early rise in CPP incidence prior to 2013, although the impact may have peaked after 2013. A trend toward earlier activation of the HPG axis in the overall Korean population may account for our recent results.

It is unclear why the onset of puberty among Korean children is accelerating. Growing research suggests that the rising prevalence of childhood obesity is a key factor in the secular trend toward earlier puberty. Several population-based longitudinal studies suggested that earlier pubertal maturation might be linked with higher adiposity in both boys and girls from many countries, including Korea [35]. The average age of menarche among Korean girls has been reduced from 13.0 years old to 12.6 years old over the last decade, and this trend was particularly pronounced in girls with overweight. Although the exact mechanisms remain to be elucidated, obesity-related hormonal changes including leptin and insulin resistance are speculated to contribute to early puberty [35]. It has been observed that obesity-related increases in circulating leptin activate hypothalamic Kiss 1 expression, which regulates GnRH pulse production and puberty onset [36]. Obesity-induced hyperinsulinemia was also shown to induce earlier pubertal onset by increasing androgen synthesis from the ovary and adrenal glands and by increasing the bioavailability of sex steroid hormones [35,37,38]. The prevalence of overweight/obesity in Korean children has rapidly increased from 15.2% to 23.7% between 2007 and 2017, and was higher in boys than in girls [39]. More pronounced increasing trends of overweight and obesity prevalence in boys compared with girls might partly explain why CPP incidence in boys has increased more rapidly than girls among Korean children. Although the findings are inconsistent, studies have also shown that low birth weight and preterm birth are associated with a faster pubertal onset or tempo than peers, especially when obesity is accompanied during childhood [40]. This phenomenon can be partly explained by increased insulin resistance in children born with low birth weight and preterm birth [41]. Between 1993 and 2016, the percentage of newborns born in Korea with low birth weight and preterm birth has increased approximately two to three times [42]. Prenatal and postnatal exposure to endocrine-disrupting chemicals (EDCs) is another frequently stated explanation for earlier pubertal onset. The relationship between EDC exposure and puberty has been explored for phenols and phthalates or polybrominated diphenyl ethers (PBDEs), although the findings of those studies have been inconsistent [43]. In Korea, cross-sectional studies reported significant associations between phthalate exposure and earlier menarche / central precocious puberty in girls, as well as between air pollution and earlier menarche; however, further studies are needed to confirm EDC exposure has contributed to the rapid increase in Korean CPP [44–46]. Furthermore, there has been a marked increase in psychological distress, a rise in screen time, a decrease in physical activity, and an increase in protein intake over the previous decades among Korean children [47,48], all of which may have influenced the advancement of pubertal tempo. However, since this was a registry-based study, we were unable to investigate the potential factors due to lack of access to individual medical records.

There are several limitations to this study. First, the etiology and epidemiologic risk factors could not be investigated since detailed medical information on variables associated with CPP, such as obesity status, family history of pubertal milestones, and the presence of organic diseases, was not included in the HIRA database. Second, patients who were diagnosed with CPP but did not receive GnRHa treatment may have been excluded in this study, which may have affected the accuracy of our study findings. Third, given that the precise dates of the pubertal onset in CPP cases were not recorded in the HIRA database, there is a possibility of overestimation of the CPP incidence, particularly among girls aged 8 years and boys aged 9 years. Lastly, we were not able to exclude unsustained CPP cases that began unnecessary GnRHa treatment. According to clinical guidelines, GnRHa treatment is not recommended for individuals with unsustained CPP that might not be linked with early menarche and short adult height.

In conclusion, our research revealed that the reported increase in CPP incidence in Korea that began in early 2000 has continued through 2020. This phenomenon was ubiquitous across all age groups and both sexes, and was not confined to older children or girls. This suggests that the onset of puberty among the general population in Korea is shifting to the left. Further research is needed to identify the precise causes of accelerated puberty onset and to establish a new pubertal timing reference in Korea.

## Supporting information

S1 Table The annual incidence of central precocious puberty according to the sex and age at diagnosis. New cases of central precocious puberty were defined as those children (boys aged 0–9 years and girls aged 0–8 years) who claimed gonadotropin-releasing hormone agonist treatment for the first time to Health Insurance Review & Assessment Service.
(DOCX)
