# Supplementary material for: Ongoing increasing trends in central precocious puberty incidence among Korean boys and girls from 2008 to 2020
Source: PLoS One. 2023 Mar 22;18(3):e0283510. doi: 10.1371/journal.pone.0283510 (PMC10032490; doi:10.1371/journal.pone.0283510)
Supplement: S1 Table — New cases of central precocious puberty were defined as those children (boys aged 0–9 years and girls aged 0–8 years) who claimed gonadotropin-releasing hormone agonist treatment for the first time to Health Insurance Review & Assessment Service. (DOCX) [file pone.0283510.s001.docx]

S1 Table. The annual incidence of central precocious puberty according to the sex and age at diagnosis

| Age | 2008 | 2009 | 2010 | 2011 | 2012 | 2013 | 2014 | 2015 | 2016 | 2017 | 2018 | 2019 | 2020 |
| --- | --- | --- | --- | --- | --- | --- | --- | --- | --- | --- | --- | --- | --- |
| Boys |  |  |  |  |  |  |  |  |  |  |  |  |  |
| 0yr | 0.0 | 0.0 | 0.0 | 0.0 | 0.0 | 0.0 | 0.0 | 0.0 | 0.0 | 0.0 | 0.0 | 0.6 | 0.0 |
| 1yr | 0.0 | 0.0 | 0.0 | 0.4 | 0.0 | 0.0 | 0.4 | 0.0 | 0.0 | 0.0 | 0.0 | 0.0 | 1.2 |
| 2yr | 0.0 | 0.4 | 0.0 | 0.4 | 0.4 | 0.0 | 0.0 | 0.0 | 0.9 | 0.0 | 0.5 | 0.0 | 0.0 |
| 3yr | 0.0 | 0.0 | 0.0 | 0.4 | 0.0 | 0.0 | 0.4 | 0.0 | 0.0 | 0.4 | 0.4 | 0.9 | 1.0 |
| 4yr | 0.0 | 0.0 | 0.0 | 0.4 | 1.2 | 0.4 | 0.4 | 0.0 | 0.8 | 1.3 | 0.0 | 0.0 | 0.5 |
| 5yr | 0.4 | 0.4 | 0.0 | 0.9 | 0.4 | 0.8 | 0.0 | 0.4 | 2.1 | 1.6 | 1.3 | 0.0 | 0.4 |
| 6yr | 0.4 | 0.4 | 0.4 | 0.9 | 0.0 | 0.4 | 0.4 | 1.7 | 1.7 | 0.8 | 3.2 | 2.5 | 4.0 |
| 7yr | 1.0 | 0.4 | 2.3 | 1.2 | 0.4 | 2.6 | 2.1 | 4.0 | 3.4 | 3.4 | 6.2 | 9.3 | 19.7 |
| 8yr | 1.5 | 3.2 | 4.7 | 5.4 | 9.2 | 14.0 | 16.6 | 16.4 | 22.6 | 38.2 | 46.0 | 69.3 | 121.1 |
| 9yr | 7.0 | 11.9 | 20.5 | 42.2 | 50.1 | 112.8 | 125.6 | 139.7 | 171.7 | 231.2 | 310.5 | 448.5 | 705.2 |
| Overall | 1.2 | 2.0 | 3.4 | 5.8 | 6.6 | 13.6 | 14.5 | 15.9 | 21.0 | 29.4 | 38.8 | 58.3 | 100.0 |
| Girls |  |  |  |  |  |  |  |  |  |  |  |  |  |
| 0yr | 0.9 | 0.0 | 1.4 | 0.0 | 0.0 | 0.0 | 0.0 | 0.0 | 0.0 | 0.0 | 0.6 | 0.0 | 0.0 |
| 1yr | 0.4 | 0.9 | 1.4 | 1.4 | 1.8 | 2.1 | 0.4 | 1.9 | 0.5 | 0.5 | 1.6 | 0.6 | 0.0 |
| 2yr | 0.9 | 1.8 | 0.9 | 2.7 | 3.6 | 1.7 | 1.7 | 2.2 | 1.9 | 2.3 | 2.4 | 3.2 | 3.6 |
| 3yr | 2.8 | 1.4 | 1.3 | 3.9 | 2.3 | 1.8 | 2.2 | 4.3 | 4.9 | 2.8 | 1.4 | 5.8 | 3.2 |
| 4yr | 3.0 | 2.3 | 4.2 | 4.4 | 3.0 | 7.7 | 7.2 | 7.8 | 7.7 | 6.6 | 9.3 | 13.0 | 13.9 |
| 5yr | 8.0 | 11.2 | 16.5 | 22.5 | 24.1 | 25.7 | 22.1 | 32.7 | 30.8 | 42.6 | 51.7 | 49.3 | 78.4 |
| 6yr | 38.1 | 52.9 | 73.6 | 99.2 | 112.5 | 118.2 | 146.7 | 127.4 | 146.6 | 188.4 | 218.4 | 258.6 | 445.2 |
| 7yr | 177.6 | 250.7 | 331.9 | 508.2 | 568.7 | 660.9 | 678.3 | 708.9 | 859.2 | 942.3 | 1175.3 | 1260.4 | 2091.1 |
| 8yr | 431.0 | 716.7 | 1030.4 | 1867.4 | 2223.8 | 2936.7 | 2977.7 | 3074.0 | 4009.9 | 4365.3 | 4892.2 | 5659.7 | 7967.3 |
| Overall | 88.9 | 135.8 | 177.8 | 291.9 | 333.6 | 406.8 | 413.1 | 449.7 | 585.4 | 633.5 | 752.0 | 911.7 | 1414.7 |
